# Suspected Agomelatine-induced restless legs syndrome: a case report

**DOI:** 10.1186/s12888-021-03175-5

**Published:** 2021-04-07

**Authors:** Mustafa Abdul Karim, Nadeen Al-Baz, Sami Ouanes, Majid Alabdulla, Peter M. Haddad

**Affiliations:** 1 Hamad Medical Corporation, Doha, Qatar; 2 Weill Cornell Medicine- Qatar, Ar-Rayyan, Qatar; 3 College of Medicine, Qatar University, Doha, Qatar

**Keywords:** Restless legs syndrome, Agomelatine, Antidepressants

## Abstract

**Background:**

Restless Legs Syndrome (RLS) is a sensorimotor disorder characterized by unpleasant and distressing sensations in the lower limbs that are more pronounced in the evening, commence or worsen at rest, and show partial or complete relief following movement. It can occur as a primary disorder, secondary to medical conditions or treatment with medications including but not limited to antidepressants or antipsychotics.

**Case presentation:**

A 32-year old man with major depressive disorder showed partial response to Escitalopram 10 mg daily. Agomelatine 25 mg at night was added to Escitalopram to treat his residual depressive symptoms, namely insomnia and tiredness. Within two days he developed restlessness and unpleasant sensations in his legs which were worse at night. Symptom severity increased over the following days, prompting an urgent consultation a week later. The patient’s presentation met the criteria for RLS. Agomelatine was discontinued leaving the patient on Escitalopram alone. The patient’s symptoms improved within 24 h of stopping Agomelatine, with complete resolution four days later. There was no recurrence of RLS during follow-up. The patient scored 6 on Naranjo’s adverse drug reaction probability scale, indicating a probable adverse drug reaction caused by Agomelatine.

**Conclusions:**

To the best of our knowledge, this is the first case report of suspected Agomelatine-induced RLS. Clinicians need to be aware of RLS to enable prompt diagnosis and management. We suggest adding Agomelatine to the list of agents that can potentially induce RLS.

## Background

Restless Legs Syndrome (RLS) or Willis-Ekbom disease is a neurological disorder characterized by an intense urge to move the lower limbs associated with unpleasant sensations that are more pronounced in the evening, commence or worsen at rest, with partial or complete relief following movement [1]. Sensations in the legs are usually described as creeping, crawling, or tingling [2, 3]. Women are disproportionally affected and age is correlated with higher occurrence in European and North American countries [4]. Higher prevalence rates are reported in western compared to Asian countries, possibly due to differences in genetics, diet and environmental factors [5].

Patients with RLS usually seek treatment due to insomnia and daytime sleepiness. Despite the name, longer duration or inadequate treatment is associated with involvement of the arms and other parts of the body [6]. Periodic Limb Movements of Sleep (PLMS) are common in patients with RLS, manifesting as involuntary contractions of the limbs and torso while awake and during sleep which differentiates it from the voluntary movements in RLS. PLMS has been reported in up to 80% of patients with RLS [5].

The most updated diagnostic criteria for RLS by the International Restless Legs Syndrome Study Group (IRLSSG) include (i) an urge to move the legs not always accompanied by unpleasant sensations (ii) beginning or worsening during periods of rest or inactivity; (iii) with partial or total relief following movement such as walking or stretching;(iv) symptoms occur or are worse during the night or evening than during the day, and (v) are not accounted for as primary to another medical condition or behavioral condition such as myalgia, venous statis, leg edema, arthritis, leg cramps, positional discomfort, or habitual foot tapping. All diagnostic criteria must be met to establish a diagnosis of RLS [1]. The subjective severity of RLS can be rated using the International Restless Legs Scale (IRLS) which comprises 10 items, each with a severity score rated over the last week on a 0 to 4 scale. The total score ranges from 0 to 40; the suggested interpretation of RLS severity is mild (0 to 10), moderate (11 to 20), severe (21 to 30) and very severe (31 to 40) [7].

RLS can be primary, secondary to a medical condition, or drug-induced. Primary or idiopathic RLS has no clearly identified cause with a gradual onset usually before 40 years of age. Family history of RLS is reported in over 50% of patients, and genome wide association studies have shown several genomic regions associated with increased incidence of the disorder [8]. Secondary RLS typically presents with sudden onset and occurs after the age of 40. RLS or RLS-like symptoms are well recognized in patients with disorders of dopamine dysfunction including Parkinson’s disease, Huntington’s disease and Tourette syndrome [9]. These findings are concordant with studies on iron-deficiency as a risk factor for the development of RLS, postulated to alter diurnal dopamine via hypoxic pathway activation [10]. Other secondary causes of RLS include pregnancy, poor cardiovascular health, hyperglycemia, impaired renal function, and diabetes mellitus [2]. Drug-induced RLS has been more commonly reported with antipsychotics, antidepressants, and antiepileptics [11]. There are currently no published case reports of Agomelatine as a potential cause of RLS. The management of secondary and drug induced RLS centers around modifying the underlying cause. Pharmacological treatment options for primary RLS include dopamine agonists and α_2_δ ligands, and the choice of drugs is based on several factors such as time of the day during which symptoms are experienced, comorbid insomnia, pain and metabolic syndromes [12].

## Case report

The patient (henceforth referred to as S) is a 32-year old North African man who was referred to the outpatient mental health services reporting low mood and insomnia. This was his first psychiatric presentation. His sister and aunt had received treatment for depression. He had no medical comorbidities and was not taking any prescribed or over-the-counter medications or supplements. He reported no history of tobacco, alcohol or illicit drug use. His intake of caffeinated beverages was limited to one or two cups of coffee in the morning. Physical examination and investigations including complete blood cell count, comprehensive metabolic panel (including liver and renal parameters), thyroid panel, vitamin D level, and electrocardiogram were unremarkable.

Clinical evaluation confirmed the diagnosis of moderate-to-severe Major Depressive Disorder (MDD) and S was prescribed Escitalopram, a selective serotonin reuptake inhibitor (SSRI) antidepressant. The initial dose was 5 milligrams (mg) daily, increased to 10 mg on the fifth day. S reported gradual improvement in appetite, mood, interest in activities and concentration. However, 3 weeks after starting Escitalopram, residual depressive symptoms, most notably insomnia and tiredness, persisted. Given this, and the fact that sleep hygiene measures had proved unsuccessful, Zolpidem 10 mg at bedtime was introduced. After 10 days, S continued to report initial and middle insomnia despite following sleep hygiene measures and adhering to medication. Agomelatine 25 mg at night was prescribed and concomitantly Zolpidem was stopped. Escitalopram was continued. Two days later, S started feeling “tingling and crawling” sensations deep in his legs before bedtime with some respite after walking or moving his legs in bed. These symptoms progressively worsened and on the seventh day of Agomelatine treatment S attended the psychiatric outpatient clinic reporting “agonizing electricity and tingling sensation running non-stop through my legs”. These symptoms were more severe at night and associated with an intense and continuous urge to walk or move the lower limbs with limited relief. Poor sleep, tiredness and low mood persisted. There was no prior history of similar symptoms. S and his partner reported no co-occurring periodic limb movements. His symptoms were construed as severe RLS, with a score of 28 on the IRLS [7]. He scored 6 on Naranjo’s adverse drug reaction probability scale, indicating a probable adverse drug reaction caused by Agomelatine [13].

**Fig. 1:**
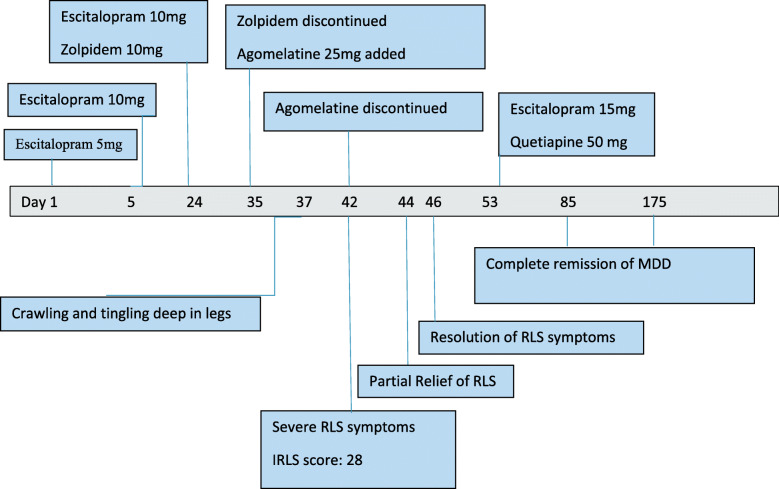
Timeline of events

Agomelatine was discontinued, and his symptoms rapidly abated from the next day onwards, completely disappearing within four days. S remained adherent to Escitalopram and free of RLS symptoms afterwards. He continued to report poor sleep patterns despite sound sleep hygiene habits along with breathing and relaxation exercises before bedtime. After discussing several treatment options, low-dose Quetiapine was commenced as an off-label treatment for his persistent insomnia. S reported a good response and no adverse effects as the dose of Quetiapine was optimized to 50 mg at bedtime and Escitalopram increased to 15 mg. On follow-up, S reported complete remission of his depressive symptoms with improved sleep quality and no RLS symptoms (Fig. 1).

## Discussion and conclusions

Agomelatine is a novel antidepressant that is an agonist at melatonin M1 and M2 receptors and a serotonin 5-HT_2c_ receptor antagonist [14]. It has been reported to increase brain-derived neurotropic factor, enhance glutamate release, and indirectly increase dopamine and norepinephrine levels in the frontal cortex. The European Medicines Agency approved Agomelatine for the treatment of MDD in 2009 after several clinical trials found it superior to placebo and as effective in treating depression as comparator antidepressants but with a favorable tolerability profile [15]. A subsequent network meta-analysis showed less dropout rates with Agomelatine compared to several other antidepressants [16]. Common adverse effects include dizziness, headache, nausea, and diarrhea with rare transaminitis and liver reactions [17]. The Summary of Product Characteristics (SPC) for Agomelatine records RLS as an uncommon neurological adverse effect. ‘Uncommon’ refers to adverse effects that occur with a frequency of between 1/1000 and 1/100 [18]. To the best of our knowledge this is the first case report of Agomelatine as a cause of drug-induced RLS.

The patient we report developed an acute onset of unpleasant sensations described as tingling, crawling and electric shock-like feelings in his legs, predominantly occurring at night and associated with an urge to move, consistent with RLS diagnosis. Agomelatine appeared to be the most likely cause; symptom emergence and resolution coincided with the initiation and discontinuation of Agomelatine and other potential causes of RLS seemed highly improbable.

Several case reports have described the emergence of RLS during Escitalopram therapy [11]. However, Escitalopram was excluded as the cause of RLS in our patient as his symptoms resolved despite continuing Escitalopram. In addition, a pharmacokinetic interaction between Agomelatine and Escitalopram is unlikely to account for the occurrence of RLS. Escitalopram is not known to induce or inhibit the activity of cytochrome P450 1A2 (CYP1A2) isoenzyme, the main metabolizer of Agomelatine [19]. Agomelatine has similarly not been shown to alter the metabolic profile of cytochrome P450 isoenzymes involved in the N-demethylation of Escitalopram (CYP2C19, CYP2D6, and CYP3A4) [20].

Differential diagnoses for RLS were considered but excluded. An important differential diagnosis was akathisia, a syndrome characterized by subjective complaints of unease and inner restlessness often leading to excessive movements including rocking and pacing. Akathisia is more commonly associated with dopamine antagonists than antidepressants [21]. It occurs shortly after commencing treatment. The SPC for Agomelatine records akathisia as a ‘rare’ neurological side effect (≥1/10,000 to < 1/1000) i.e. according to the SPC it is a less common adverse effect than RLS [18]. In akathisia, the urge to move is not limited to the lower limbs, lacks a circadian pattern and is less likely to be associated with paresthesia than RLS [22]. In summary, the symptoms profile in our patient was more consistent with RLS than akathisia.

Another differential is serotonin syndrome, or serotonin toxicity, a potentially life-threatening disorder precipitated by serotonergic agents that can emerge with antidepressant combination. Signs and symptoms include hypertension, tachycardia, mydriasis, diaphoresis, hyperactive bowel sounds, clonus, agitation, rigidity, hyperreflexia, and hyperthermia [23]. It is unlikely that our patient suffered from serotonin syndrome as he was vitally stable with an unremarkable physical examination. Furthermore, serotonin syndrome is usually associated with combinations of drugs that increase serotonin transmission through different pathways [23]. The combination of agomelatine and escitalopram is unlikely to have caused serotonin syndrome as agomelatine has no effect on serotonin levels, rather it acts on melatonin, noradrenaline and dopamine pathways [14]. Myalgia and nocturnal leg cramps were excluded as the patient’s sensations were not described as cramping-type or tightening of the lower limb muscles. Past medical history, physical examination and investigations were unremarkable, excluding RLS secondary to specific physical illnesses.

Zolpidem withdrawal was considered given that our patient’s symptoms appeared two days after Zolpidem was discontinued. However, Zolpidem withdrawal can be excluded as our patient was prescribed Zolpidem for only 10 days and at a therapeutic dose. Neither the duration nor the dose of use are consistent with the occurrence of Zolpidem withdrawal syndrome. Indeed, Zolpidem administration at therapeutic doses for 12 months has not been shown to significantly increase the likelihood of rebound insomnia or withdrawal symptoms [24, 25]. Most reported cases of Zolpidem withdrawal have been associated with abuse where individuals took Zolpidem at exceedingly large doses or for prolonged periods [26–30].

The pharmacodynamic underpinnings of RLS in this case are speculative. Decreased dopaminergic neurotransmission appears to be involved in the pathophysiology of RLS [31]. In theory, Agomelatine should have a low risk for inducing RLS as it increases fronto-cortical dopamine transmission secondary to 5-HT_2C_ receptor antagonism [32]. Michaud et al analyzed several factors associated with the circadian rhythm of symptom intensity in RLS and found subjective vigilance, core body temperature, and salivary melatonin level positively correlated with profiles of leg discomfort. Of those, only melatonin changes preceded the increase in symptom intensity [33]. This raises the possibility that Agomelatine could potentially provoke RLS through its action as a melatonin MT1 and MT2 receptor agonist.

It is notable that our patient suffered from depression and RLS. Gupta et al’s cross-sectional assessment found a 31.5% prevalence of RLS among patients diagnosed with MDD [34]. A high rate of depression has also been reported in patients with RLS, although prevalence rates seem to be related to RLS severity [35, 36]. The relationship between RLS and depression is complex; it may be bidirectional and reflect common pathophysiological factors. Dysregulated central dopaminergic neurotransmission has been elicited in both depression and RLS [5, 37]. In addition, insomnia and subsequent daytime sleepiness and fatigue overlap between both disorders. Antidepressants also have the potential to worsen RLS symptoms, further complicating the treatment of depression with comorbid RLS. Of note, RLS induced by Mirtazapine treatment, a tetracyclic antidepressant, has been reported with a higher prevalence compared to other antidepressants including SSRIs [38, 39].

The patient we report was highly distressed by his symptoms. This is consistent with literature highlighting severe RLS as a debilitating disorder affecting patients, partners and family members [40]. A multicenter study reported that health-related quality of life in patients with RLS is significantly lower compared to the general population and comparable to patients with chronic neurological disorders such as Parkinson’s disease and stroke [41]. This is in line with a recent cross-sectional study that showed an association between RLS and sleep disturbance, excessive daytime sleepiness and reduced physical quality of life [42].

RLS is a distressing syndrome that can occur either as a primary disorder or secondary to specific physical disorders or various medications including antipsychotics and antidepressants. An important differential diagnosis is akathisia. Clinicians need to be familiar with RLS to allow prompt diagnosis and treatment. Based on this report, Agomelatine should be added to the list of agents that can potentially induce RLS. Further research, including post marketing surveillance, is necessary to determine the relative risk of RLS with Agomelatine versus other antidepressants.

## Data Availability

Not applicable.

## Abbreviations

CYP: Cytochrome P450
IRLS: International Restless Legs Scale
IRLSSG: International Restless Legs Syndrome Study Group
MDD: Major Depressive Disorder
PLMS: Periodic Limb Movements of Sleep
RLS: Restless Legs Syndrome
SPC: Summary of Product Characteristics
